# Crosstalk Between Four Types of RNA Modification Writers Characterizes the Tumor Immune Microenvironment Infiltration Patterns in Skin Cutaneous Melanoma

**DOI:** 10.3389/fcell.2022.821678

**Published:** 2022-01-26

**Authors:** Shichao Zhang, Yu Xiong, Chaochao Zheng, Jinhua Long, Houming Zhou, Zhu Zeng, Yan Ouyang, Fuzhou Tang

**Affiliations:** ^1^ Key Laboratory of Infectious Immune and Antibody Engineering in Guizhou Province, School of Biology and Engineering, Guizhou Medical University, Guiyang, China; ^2^ Immune Cells and Antibody Engineering Research Center of Guizhou Province, School of Biology and Engineering, Guizhou Medical University, Guiyang, China

**Keywords:** RNA modification “writer”, skin cutaneous melanoma, tumor microenvironment, W_Score, immunotherapy

## Abstract

The “writers” of four types of adenosine (A)-related RNA modifications (N6-methyladenosine, N1-methyladenosine, alternative polyadenylation, as well as A-to-inosine RNA editing) are closely related to the tumorigenesis and progression of many cancer types, including skin cutaneous melanoma (SKCM). However, the potential roles of the crosstalk between these RNA modification “writers” in the tumor microenvironment (TME) remain unclear. The RNA modification patterns were identified using an unsupervised clustering method. Subsequently, based on differentially expressed genes responsible for the aforementioned RNA modification patterns, an RNA modification “writer” scoring model (W_Score) was constructed to quantify the RNA modification-associated subtypes in individual patients. Moreover, a correlation analysis for W_Score and the TME characteristics, clinical features, molecular subtypes, drug sensitivities, immune responses, and prognosis was performed. We identified three RNA modification patterns, corresponding to distinct tumor immune microenvironment characteristics and survival outcomes. Based on the W_Score score, which was extracted from the RNA modification-related signature genes, patients with SKCM were divided into high- and low-W_Score groups. The low-W_Score group was characterized by better survival outcomes and strengthened immunocyte infiltration. Further analysis showed that the low-W_Score group was positively associated with higher tumor mutation burden and PD-L1 expression. Of note, two immunotherapy cohorts demonstrated that patients with low W_Score exhibited long-term clinical benefits and an enhanced immune response. This study is the first to systematically analyze four types of A-related RNA modifications in SKCM, revealing that these “writers” essentially contribute to TME complexity and diversity. We quantitatively evaluated the RNA modification patterns in individual tumors, which could aid in developing personalized immunotherapy strategies for patients.

## Introduction

Worldwide, there were 324,635 and 57,043 estimated new skin cutaneous melanoma (SKCM) cases and deaths, respectively, in 2020 (Sung et al., 2021). SKCM accounts for approximately 4% of all cases of skin cancer and is the most fatal subtype of skin cancer (Lin et al., 2021). Previous studies have demonstrated that the occurrence and development of SKCM are related to the accumulation of mutations in gene-modulating signaling pathways, including the Rb, p53, PI3K/AKT, and RAS/MAPK pathways (Dietrich et al., 2018; Chamcheu et al., 2019). Although the onset of SKCM may be partially attributed to somatic mutations, epigenetic changes in cancer-related genes are also associated with its etiology (de Unamuno Bustos et al., 2018). Epigenetic alterations mainly affect the functions and characteristics of genes by regulating gene transcription or translation (Bauer et al., 2016; Gu H. et al., 2020; Blanco et al., 2021). Furthermore, increasing evidence has revealed that RNA modification is mechanism that is indispensable for epigenetic regulation, which is involved in tumorigenesis and development of multiple cancers, including SKCM (Gu et al., 2015; Shen et al., 2019).

Over 170 types of RNA modifications have been identified (Wang et al., 2013; Barbieri and Kouzarides, 2020; Chen et al., 2021). Some of these modifications may interact to play key roles in many important biological processes, but synthetically analyzing all types of RNA modifications is difficult. Adenosine (A) is the most commonly modified nucleotide in RNA, with modifications that include N1-methyladenosine (m1A), N6-methyladenosine (m6A), and A-to-inosine (I) (Chen et al., 2021). Particularly, the m6A methylase negatively modulates the A-to-I RNA editing (Xiang et al., 2018). Thus, in this work, we focused on A-associated RNA modifications [m1A, m6A, A-to-I, and alternative polyadenylation (APA)] to explore the interaction by the “writers” that produced these modifications.

m6A is the most common RNA modification. Till date, this modification has been found in mRNAs, lncRNAs, miRNAs, circRNAs, rRNAs, tRNAs, and snRNAs (Zhao et al., 2017; Gu L. et al., 2020). Methyltransferases catalyze m6A methylation, and these “writers” include METTL14, METTL3, RBM15, RBM15B, VIRMA, WTAP, and ZC3H13 (Cao et al., 2016). In particular, m6A regulators with abnormal expression and genetic changes are involved in the pathogenesis and development of tumors, as well as in immune dysregulation (Li et al., 2017; Xiang et al., 2017; Zhang et al., 2017; Li et al., 2019; Gu C. et al., 2020). m1A is a methylation modification at the first nitrogen atom of the A base of the RNA molecule, which exists in tRNA, rRNA, and mRNA (Agris, 1996; Safra et al., 2017). The “writers” of the m1A modification include TRMT6, TRMT10C, TRMT61A, and TRMT61B (Dominissini et al., 2016; Wang Y. et al., 2019). Similar to the m6A modification, A-to-I RNA editing is also a prevailing RNA modification in the A base of mRNA (Di Giammartino et al., 2011; Abudayyeh et al., 2019). A-to-I RNA editing is mainly mediated by members of the A deaminase in the RNA (ADAR) family, including ADARB1, ADARB2, and ADAR, which bind to double-stranded RNA regions of protein-coding genes and non-coding sequences for the deamination of A to I (Huang et al., 2012; Han et al., 2014). APA is an important precursor-RNA processing mechanism widely present in all eukaryotes, which can regulate the length of the 3′-untranslated region (3′UTR) by cleaving mRNA at different sites, followed by the addition of poly(A) tails to the RNA 3′UTR (Elkon et al., 2013; Masamha et al., 2014). Several APA-related “writers” have been found to modulate the synthesis of poly (A) tails and the selection of variable poly (A) sites, including CFI, CLP1, CPSF1, CPSF2, CPSF3, CPSF4, CSTF1, CSTF2, CSTF3, NUDT21, PABPN1, and PCF11 (Batra et al., 2015).

The crosstalk among m1A, m6A, A-to-I, and APA can help reveal the mechanism underlying RNA modifications and the significance of post-transcriptional modifications. In SKCM, a complex regulatory network may be formed by the “writers” of the m1A, m6A, A-to-I, and APA modifications. Therefore, systematic analysis of the interaction between these “writers” could provide novel insights into the pathogenesis of SKCM and has potential clinical significance for tumor therapeutic target identification.

Current immunotherapies based on immune-checkpoint inhibitors (ICIs) have achieved astounding clinical efficacy in some patients. Unfortunately, immunotherapy is not effective in approximately 40–50% of patients with SKCM (Albittar et al., 2020). Tumor cells in the tumor microenvironment (TME) interact directly and indirectly with other components to induce hypoxia, chronic inflammation, and immunosuppression. Indeed, the abundance of tumor-infiltrating immune cells has been found to be associated with the prognosis of patients with SKCM (Ladányi et al., 2004). A high proportion of infiltrating CD8^+^ T cells appears to be a more effective immunotherapeutic response (Daud et al., 2016). Base on the characteristics of TME, tumors can be classified into immune-desert, immune-excluded, and immune-inflamed phenotypes, which present differences in the number of infiltrating immune cells and the immunotherapy response (Chen and Mellman, 2017; Zhang et al., 2020).

Recent studies have showed that the RNA modification “writers” are closely related to TME immune cell infiltration. Gao et al. revealed that m1A regulators, including “writers”, modulated the infiltration of immune cells in TME (Gao et al., 2021). Wang et al. found that m6A modification mediated by the RNA methyltransferase METTL3 promoted the functional activation of dendritic cells (DCs). Silencing METTL3 significantly downregulated the m6A modification levels, leading to a decreased antigen presentation ability of mature DCs (Wang H. et al., 2019). These studies were limited to one type of RNA modification, but multiple types of “writers” exert antitumor effects in a highly coordinated manner in cancer. Thus, systematic identification of the correlation between regulatory networks composed of multiple types of “writers” and the TME will be helpful in predicting immunotherapy responses and developing new immunotherapy strategies.

In this study, we comprehensively analyzed the relationship between four types of A-related RNA modification patterns and the characteristics of infiltrating immune cells by integrating transcriptomic data from 1526 SKCM samples from the Genotype-Tissue Expression (GTEx), Gene Expression Omnibus (GEO), and The Cancer Genome Atlas (TCGA) databases. Three distinct RNA modification patterns were identified, and the characteristics of the TME in these three patterns corresponded to immune-desert phenotype, immune-excluded phenotype, and immune-inflamed phenotype, respectively. We further constructed an RNA modification “writers” scoring model to quantify RNA modifications patterns in individual patients and to evaluate the patient response to targeted therapy and immunotherapy.

## Methods

### Data Acquisition of Skin Cutaneous Melanoma Samples

The workflow of this study is presented in Supplementary Figure S1A. Large-scale transcriptome data (including normal and tumor samples) was downloaded from the TCGA (https://tcga-data.nci.nih.gov/tcga/), GTEx (https://gtexportal.org/), and GEO (http://www.ncbi.nlm.nih.gov/geo) databases. Clinical information (including tumor stage, gender, age, and overall survival times) and somatic mutation and copy number variation (CNV) data from corresponding patients were retrieved from the University of California Santa Cruz genome browser (http://genome.ucsc.edu/). Patients with overall survival times <90 days and without survival information were excluded. Two GEO-SKCM cohorts (GSE78220 and GSE65904), one TCGA-SKCM cohort, and one GTEx-SK cohort were obtained for further analysis. The “limma with normalizeBetweenArrays” package was used to correct for batch effects of non-biotechnology deviation, and the basic information of these datasets is shown in Supplementary Table S1.

### Unsupervised Clustering and Differential Expression Analysis of 26 “Writers”

Twenty-six RNA modification “writers” were obtained from the TCGA-SKCM cohort for identifying distinct RNA modification patterns. These 26 RNA modification “writers” included seven m6A regulators (METTL14, METTL3, RBM15, RBM15B, VIRMA, WTAP, and ZC3H13), four m1A regulators (TRMT6, TRMT10C, TRMT61A, and TRMT61B), three A-I regulators (ADARB1, ADARB2, and ADAR), and 12 APA regulators (CFI, CLP1, CPSF1, CPSF2, CPSF3, CPSF4, CSTF1, CSTF2, CSTF3, NUDT21, PABPN1, and PCF11). Based on the expression of 26 RNA modification “writers”, the tumor samples were classified (defined as “writer cluster”) *via* the unsupervised clustering analysis (Wilkerson and Hayes, 2010). For guaranteeing clustering stability, 1,000 iterations were performed with Spearman distance and pltem = 0.8 using the PAM algorithm (“ConsensuClusterPlus” package). The Wilcoxon signed-rank test was employed to analyze the differential expression of the 26 RNA modification “writers” between normal and tumor samples, and the expression correlation between these regulators was identified using Pearson’s correlation analysis.

### Gene Set Variation Analysis and Functional Annotation

To examine the variations in RNA modification patterns in biological processes, gene set variation analysis (GSVA enrichment analysis; using the “GSVA” R package) was conducted (Hänzelmann et al., 2013). Gene sets with “c2.cp.kegg.v7.4.-symbols-gmt” and “c5.go.bp.v7.4.symbols.gmt” were retrieved from the Molecular Signatures Database followed by GSVA analysis with an adjusted *p* < 0.05. The functional annotation for 26 RNA modification “writers” was performed using the “clusterProfiler” R package (Yu et al., 2012).

### Evaluation of Tumor-Infiltrating Immune Cells in the Tumor Microenvironment

Single-sample gene set enrichment analysis (ssGSEA) was employed to evaluate the relative proportion of each infiltrating immune cell type in the SKCM TME (Barbie et al., 2009; Charoentong et al., 2017), including adaptive immune cells (activated B cells, activated CD4^+^ T cells, activated CD8^+^ T cells, gamma delta T cells, immature B cells, regulatory T cells, T follicular helper cells, type 1 T helper cells, type 17 T helper cells, and type 2 T helper cells) and innate immune cells (activated DCs, CD56bright natural killer cells, CD56dim natural killer cells, eosinophils, immature DCs, macrophages, mast cells, MDSCs, monocytes, natural killer cells, natural killer T cells, neutrophils, and plasmacytoid DCs) (Supplementary Table S2). The enrichment scores were also calculated *via* ssGSEA.

### Association Between Clusters and Other Biological Processes

Correlation analysis between categories and some associated biological processes was conducted. According to previous reports, gene sets including several gene-related biological processes were constructed, and these biological pathways included antigen processing and presentation, DNA damage repair, pan-fibroblast TGF-β response signature (pan-F-TBRS), angiogenesis, DNA replication, nucleotide excision repair, mismatch repair, WNT targets, cell cycle, CD8^+^ T effectors, antigen processing machinery, and immune-checkpoint genes (Şenbabaoğlu et al., 2016; Mariathasan et al., 2018).

### Identification of Differentially Expressed Genes Between Different RNA Modification Phenotypes

To identify differentially expressed genes (DEGs) among the different RNA modification phenotypes (three distinct RNA modification patterns were identified based on the aforementioned unsupervised clustering analysis), an empirical Bayesian approach in the “limma” R package with an adjusted *p* < 0.05 was adopted (Ritchie et al., 2015). Furthermore, for functional annotation, we used the “clusterProfiler” R package to analyze DEGs with *p* < 0.05.

### Constructing Gene Clusters

A univariate Cox regression analysis was performed to obtain prognosis-related DEGs, and unsupervised clustering analysis was then employed to classify these genes. In the PAM algorithm (using the “ConsensuClusterPlus” package) (Wilkerson and Hayes, 2010), 1,000 iterations were performed with Pearson distance and pltem = 0.8 to ensure the stability of the clusters.

### Establishment of the W_Score Scoring System

Principal component analysis was used to construct the W_Score scoring model. First, we established a matrix consisting of expression levels of the prognosis-related DEGs of each patient. Then, principal components 1 and 2 were both selected act as signature scores. Finally, we calculated the W_Score for each cancer patient (similar to GGI) (Sotiriou et al., 2006):

$$\mathrm{W–Score=}\sum PC1_{i}+PC2_{i}$$

where *i* denotes the expression of prognosis-related DEGs. Finally, according to the cutoff value (−0.646) determined using the “survminer” package, the patients with SKCM were divided into two categories (high W_Score and low W_Score).

### Association Between W_Score and Immune-Related Signal Pathways

Correlation analysis between the W_Score and potential biological processes was conducted based on gene sets, including antigen processing and presentation, DNA damage repair, pan-F-TBRS, angiogenesis, DNA replication, nucleotide excision repair, mismatch repair, WNT targets, cell cycle, CD8^+^ T effectors, antigen processing machinery, immune-checkpoint, apoptosis regulation, ABL signaling, cytoskeleton, mitosis, ERK/MAPK signaling, RTK signaling, PI3K/MTOR_signaling, p53 pathway, protein stability and degradation, IGF1R signaling, genome integrity, JNK and p38 signaling, chromatin histone acetylation, EGFR signaling, metabolism, and WNT signaling pathways (Supplementary Table S6) (Iorio et al., 2016; Şenbabaoğlu et al., 2016; Mariathasan et al., 2018).

### Evaluation of W_Score Correlations With Clinical Features and Drug Sensitivity

We evaluated the relationship between the clinicopathological characteristics, such as the Clark levels (I, II, III, IV, and V grades), transcriptomic classifications (immune, keratin, and MITF-low), and mutations subtypes (BRAF, RAS, and NF1) (Network, 2015), and the W_Score using the Wilcoxon signed-rank test. In addition, the data on drug response and targets/pathways were obtained from the Genomics of Drug Sensitivity in Cancer (GDSC) (http://www.cancerrxgene.org/downloads) (Supplementary Table S7) (Yang et al., 2013), and the Spearman’s correlation analysis was used to analyze the correlation between the W_Score and drug sensitivity. The “pRRophetic” package was used with an |Rs| > 0.2.

### Analysis of W_Score and Immune-Checkpoint Genes

Gene expression profiles of the immune-checkpoint in patients with SKCM were retrieved from The Cancer Immunome Database (TCIA) (http://tcia.at/home) (Charoentong et al., 2017), including CTAL-4 positive and PD-1 negative (ips_ctla4_pos_pd1_neg), CTAL-4 positive and PD-1 positive (ips_ctla4_pos_pd1_pos), and CTAL-4 negative and PD-1 positive (ips_ctla4_neg_pd1_pos). Two tumor immunotherapeutic cohorts, including advanced urothelial cancer (treatment with atezolizumab, an anti-PD-L1 antibody) (Mariathasan et al., 2018), and metastatic melanoma (treatment with pembrolizumab, an anti-PD-1 antibody) (Hugo et al., 2016), were downloaded from http://research-pub.gene.com/IMvigor210CoreBiologies and GEO (GSE78220), respectively.

### Statistical Analysis

The differential expression analysis of 26 RNA modification “writers” between normal and tumor samples was conducted using the Wilcoxon signed-rank test. The correlation coefficients of the expression of 26 RNA modification “writers” were investigated using Pearson’s correlation analysis, while the correlation coefficients between tumor-infiltrating immune cells and 26 RNA modification “writers” were evaluated using the Wilcoxon signed-rank test. The hazard ratios (HRs) of RNA modification “writers” and DEGs were separately calculated *via* univariate Cox regression model. We used the Kaplan-Meier method with log-rank tests to obtain survival curves, and the reliability of the models was analyzed by the receiver operating characteristic (ROC) curve. A genome-wide CNV map with the 26 RNA modification “writers” was drawn using the R package “RCircos” (Mayakonda et al., 2018). All statistical analyses were two-sided, and *p* < 0.05 was considered statistically significant.

## Results

### Genetic Variation of Four Types of A-Associated RNA Modification “Writers” in SKCM

**FIGURE 1:**
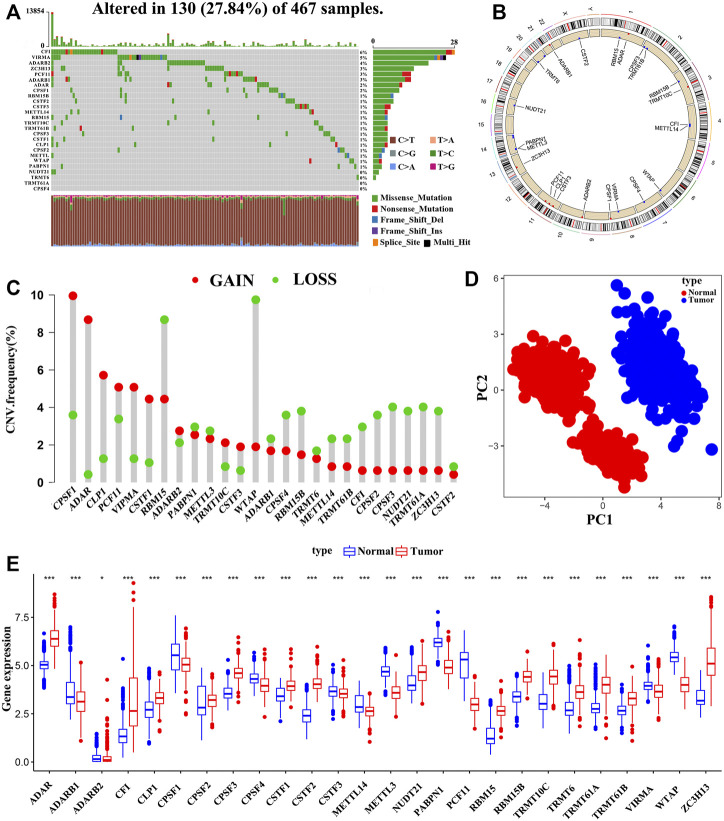
The Genomic alterations and aberrant expression of RNA modification “writers” in SKCM. **(A)** The frequency of mutation of 26 RNA modification “writers” in 467 samples from the TCGA-SKCM cohort. **(B)** The location of copy number variation (CNV) of 26 RNA modification “writers” on 23 chromosomes. **(C)** The CNV frequency of 26 RNA modification “writers” in TCGA-SKCM cohort. **(D)** Principal component analysis based on the expression of 26 RNA modification “writers” to distinguish tumors (red dots) from normal tissues (blue dots) in the TCGA-SKCM and GTEx-SK cohorts. **(E)** The expression differences of 26 RNA modification “writers” between normal and tumor tissues. Tumor, red; Normal, blue. (*p < 0.05; **p < 0.01; ***p < 0.001).

In this study, 26 A-associated RNA modification “writers”, including seven m6A modifications “writers”, four m1A modifications “writers”, three A-I modifications “writers”, and twelve APA modifications “writers”, were finally identified. We first evaluated the incidence of somatic mutations of the 26 RNA modification regulators in SKCM. Among the 467 samples, a mutation in at least one “writer” was observed in 130 patients (mutation frequency, 27.84%) (Figure 1A). Among the 26 “writers,” CFI exhibited the highest mutation frequency (6%), followed by VIRMA (5%) and ADARB2 (4%), whereas no mutations were found in TRMT61A and CPSF4 (Figure 1A). Notably, a significant co-occurrence between ZC3H13 and TRMT10C, ZC3H13 and CSTF1, ZC3H13 and ADAR, ADARB2 and RBM15, and VIRMA and PCF11 was observed (Supplementary Figure S2A). Furthermore, the patients were divided into two groups (the “writers” mutation group and the non-mutation group), and enrichment analysis *via* GSVA revealed that tumor hallmarks associated gene sets, including the KRAS signaling pathway, DNA repair pathway, and the PI3K/AKT/MTOR signaling pathway, were mainly enriched in “writers” mutation group (Supplementary Figure S2C). It has been demonstrated previously that the PI3K/AKT/MTOR signaling pathway is actively involved in the regulation of cancer cell proliferation, metastasis, survival, as well as the angiogenesis (Chamcheu et al., 2019). These findings suggested that mutations in A-associated RNA modification “writers” may cause functional changes thereby affecting SKCM progression.

We also investigated the CNVs of these “writers” and found that somatic copy number alterations were widespread in the 26 RNA modification regulator (Figure 1C). CPSF1 exhibited the highest frequency of CNV gains, followed by ADAR and CLP1, whereas CNV loss was observed in WTAP. Figure 1B showed the 26 RNA modification “writers” loci on a schematic of the genome. Interestingly, based on the expression levels of the 26 “writers”, the normal and tumor samples could be completely distinguished by principal component analysis (Figure 1D). We further discovered that the 26 RNA modification regulators have heterogeneous expression between normal and tumor tissues (Figure 1E), and the “writers” with amplified CNVs presented markedly higher expression in SKCM tissues, suggesting that CNV is one of the main factors that regulate “writers” expression (Supplementary Figure S3). The above analysis indicates that these RNA modification regulators with genomic alterations and expression imbalance play a potential role in the onset and development of SKCM.

### Correlation Between RNA Modification “Writers” and TME Characteristics

**FIGURE 2:**
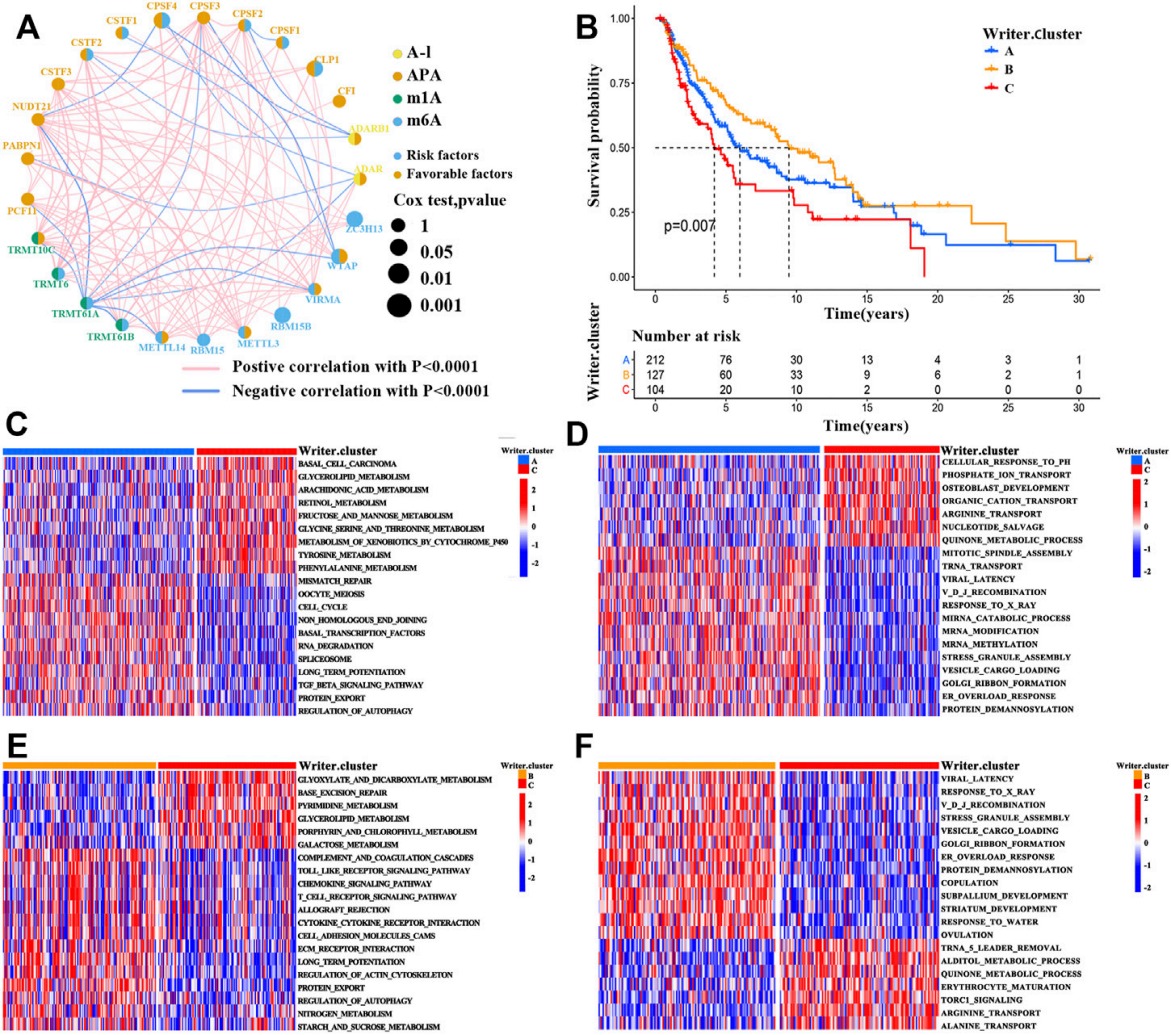
RNA modification patterns and corresponding biological characteristics of each pattern. **(A)** The correlations between 26 RNA modification “writers” expression in SKCM. The circle size represented the prognostic value of each regulator. **(B)** Survival analyses for the three RNA modification patterns based on 443 patients with SKCM from TCGA-SKCM cohort (Log-rank test; *p* = 0.007). **(C–F)** Comparison of GSVA and GO enrichment analysis (biological pathways) in distinct RNA modification patterns. **(C,E)**, GSVA enrichment analysis; **(D,F)**, GO enrichment analysis; **(C,D)**, writer cluster A vs. writer cluster B; **(E,F)**, writer cluster B vs. writer cluster C.

The comprehensive landscape of the interactions of 26 RNA modification “writers” and their prognostic values in patients with SKCM was described in the regulator network (Figure 2A and Supplementary Figure S2B and Supplementary Table S3). We found that the expression of RNA regulators among the different types of RNA modification showed significant correlations. Therefore, crosstalk among these writers may play critical roles in the formation of distinct RNA modification patterns.

Based on the expression of the RNA modification “writers’’, unsupervised clustering analysis was then employed to stratify SKCM tumors with qualitatively different RNA modification patterns (Supplementary Figure S6A-D). A total of 3 clusters with distinct modification patterns were obtained, including 212 patients in cluster 1 (writer cluster A), 127 patients in cluster 2 (writer cluster B), and 103 patients in cluster 3 (writer cluster C). Next, based on the prognostic analysis for these clusters, patients in cluster_writer B were found to have a prominent survival advantage, whereas a poor prognosis was revealed for patients in writer cluster C (Figure 2B; log-rank test, *p* = 0.007). To further explore the biological significance underlying these distinct RNA modification patterns, GSVA enrichment analysis was conducted. The writer cluster A was significantly related to carcinogenic activation pathways (e.g., the TGF-β signaling pathway, the JAK-STAT signaling pathway, and the regulation of autophagy) (Figure 2C). The writer cluster B was mainly enriched in immune-associated signaling pathways (e.g., allograft rejection, chemokine signaling pathways, toll-like receptor signaling pathway, cytokine–cytokine receptor interactions, and T cell receptor signaling pathways) (Figure 2E), while writer cluster C was enriched in signaling pathways associated with metabolic reprogramming (e.g., tyrosine metabolism, glycine serine and threonine metabolism, and phenylalanine metabolism) (Figures 2C,E). These results were supported by gene ontology (GO; biological process) enrichment analysis (Figures 2D,F).

**FIGURE 3:**
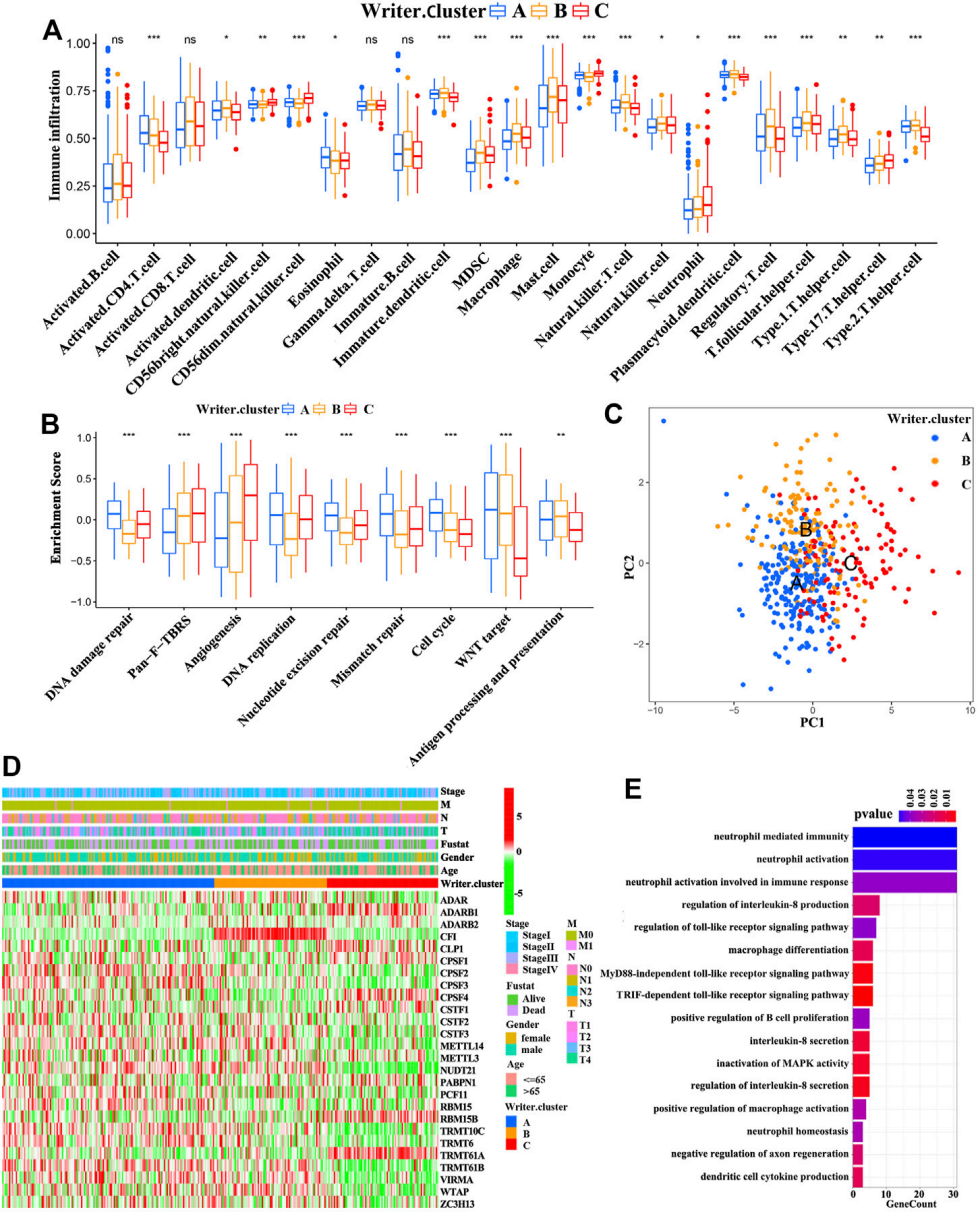
TME and transcriptome characteristics in distinct RNA modification patterns. **(A)** Evaluating the abundance of each tumor-infiltrating immune cell in three writer clusters using ssGSEA. (*p < 0.05; **p < 0.01; ***p < 0.001). **(B)** Differences in immune-associated pathways among three writer clusters (ANOVA analysis; *p < 0.05; **p < 0.01; ***p < 0.001). **(C)** Principal component analysis for the transcriptome profiles of patients in different writer clusters. **(D)** The association between RNA modification patterns (based on writer clusters) and clinical parameters, including age, gender, clinical stage, T-stage, N-stage, M-stage and survival status. **(E)** Functional annotation for differentially expressed genes (DEGs) (GO enrichment analysis, biological progress; BP).

Because patients with writer cluster B presented a remarkable survival advantage and showed enrichment of immune-related pathways, we attempted to examine the functional roles of distinct RNA modification patterns in TME. The results showed that in the TCGA cohort (Supplementary Table S4), the writer cluster B exhibited increased fractions of tumor-infiltrating immune cells (e.g., mast cells, macrophages, MDSCs, plasmacytoid DCs, activated DCs, immature DCs, and natural killer cells) (Figure 3A), as well as a higher enrichment score for immune-related pathways (e.g., antigen processing and presentation) (Figure 3B), which was similar to the findings obtained for the GEO cohort (GSE65904; Supplementary Figure S4). Thus, writer cluster B could be characterized as an immune “hot” and activation phenotype, while writer cluster C was an immune “cold” and desert phenotype (Figures 3A,B). Previous studies have demonstrated that the prognosis for patients with SKCM is positively related to tumor-infiltrating immune cells, such as MDSCs, DCs, and natural killer cells, whereas it is negatively correlated with the infiltration of neutrophils and monocytes (Schmidt et al., 2007; Chevolet et al., 2015; Cursons et al., 2019), which is consistent with our results.

Next, Spearman’s correlation analysis was conducted to investigate the specific associations between infiltrating immune cells and RNA modification “writers” (Supplementary Table S5A). We observed that WTAP (an m6A modification “writer”) demonstrated a positive correlation with a large number of TME infiltrating immune cells in SKCM. In particular, patients with elevated expression of WTAP presented remarkable more enrichment of TME DCs infiltration, including plasmacytoid DCs, activated DCs and immature DCs (Supplementary Figure S5B-C). Recently, Cao et al. proposed that RNA modulation “writers” can promote DC activation. DCs acting as antigen-presenting cells are a bridge between innate and adaptive immunities, and the activation of DCs relies on the high expression of adhesion factors, costimulatory factors, and MHC molecules (Qian and Cao, 2018). As expected, patients with a high expression of WTAP had an overall increase in the expression of adhesion factors, costimulatory factors, and MHC molecules (Supplementary Figure S5D). We also noted that the enhancement of immune-related pathways is accompanied by elevated expression of immune-checkpoint PD-1/L1 (CD279/CD274) in tumors with high expression of WTAP (Supplementary Figure S5D-E). Therefore, we evaluated the therapeutic effects of immune-checkpoint blocking antibodies between high and low WTAP expression patients. In anti-PD-1 immunotherapy cohort (GSE78220; Patients with metastatic melanoma treated with anti-PD-1 antibody immunotherapy), patients with a high expression of WTAP exhibited a survival benefit trend (Supplementary Figure S5F). Interestingly, WTAP was also associated with prolonged survival in GEO cohort (GSE65904; Supplementary Figure S5G). Taken together, WTAP may regulate the activation of DCs in the TME *via* RNA modification, participating in the antitumor immune response.

### RNA Modification “Writer” Phenotype-Related DEGs

**FIGURE 4:**
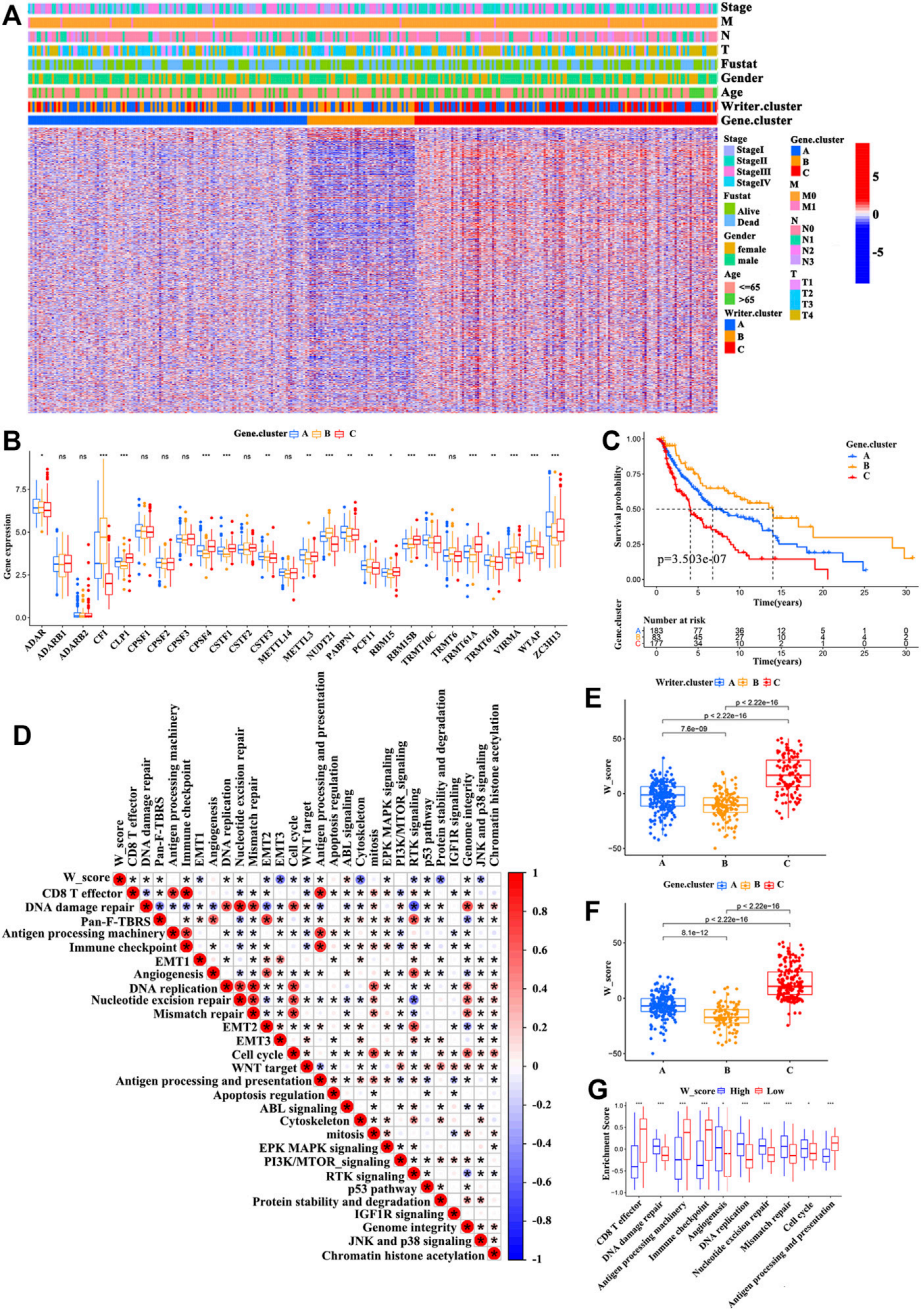
Construction of the W_Score. **(A)** Unsupervised clustering of RNA modification phenotype-related differentially expressed genes (DEGs) in TCGA-SKCM cohort to classify patients into different genomic subtypes, defined as gene cluster A, B, and C. The writer clusters, gene clusters, age, gender, clinical stage, T-stage, N-stage, M-stage and survival status were used as patient annotations. **(B)** The differences in expression of 26 A-related RNA modification “writers” in three gene clusters (TCGA-SKCM cohort). (ANOVA analysis; *p < 0.05; **p < 0.01; ***p < 0.001). **(C)** The survival curves of three gene clusters based on 443 patients from TCGA-SKCM cohort (Log-rank test; *p* < 0.001). **(D)** The associations between W_Score and the known signaling pathways in TCGA-SKCM cohort. **(E,F)** Differences of the W_Score in writer clusters **(E)** and gene clusters **(F)**, respectively, in TCGA-SKCM cohort (K-W test; *p* < 0.001). **(G)** Differences in immune-related pathways between high- and low- W_Score groups. (*p < 0.05; **p < 0.01; ***p < 0.001).

Despite patients with SKCM could be divided into three RNA modification phenotypes based on the expression of the RNA modification “writers” (Figures 3C,D and Supplementary Figure S6E), the genomic alterations and expression profile differences in these phenotypes are unclear. Therefore, we analyzed the changes in RNA modification-associated transcriptional expression across three RNA modification patterns. A total of 2281 RNA phenotype-associated DEGs were identified using an empirical Bayesian approach in the “limma” package (Supplementary Figure S6F). The “clusterProfiler” package was then used to perform GO analysis for these DEGs, and it showed that the biological processes were related with neutrophil-mediated immunity signaling pathway, neutrophil activation involved in immune response signaling pathway, regulation of the toll-like receptor signaling pathway, and the TRIF-dependent toll-like receptor signaling pathway (Figure 3E). 950 of these DEGs were significantly correlated with clinical outcomes, which were considered as RNA modification-associated signature genes. These results reconfirmed that RNA modification “writers” played an important role in the immune regulation in TME. Unsupervised clustering analysis was conducted to further verify this modulation mechanism, based on the gained 950 RNA phenotype-associated signature genes. This analysis classified patients into three genomic subtypes (Supplementary Figure S7A-D and Figure 4A), defined as gene cluster A, gene cluster B, and gene cluster C (183 cases, 83 cases, and 177 cases were clustered into gene cluster A, gene cluster B, and gene cluster C, respectively). We observed that the survival probability of patients was significantly different among these categories, and patients in gene cluster B presented the best prognosis compared with patients in gene cluster B and gene cluster C (with patients in gene cluster C showing the worst survival outcomes) (Figure 4C). Furthermore, a significant difference among the three categories was found for a variety of immune-related marker genes and enriched signaling pathways as well as the expression levels of 26 RNA modification “writers” (Figure 4B, and Supplementary Figure S7E-I). In addition, differences in prognosis and RNA “writer” expression between the three subgroups were further confirm by the analysis of patients with SKCM from GEO (GSE65904) (Supplementary Figure S8A-B). To our surprise, the characteristics of prognosis and the TME of gene cluster A, B, and C correspond to writer cluster A, B, and C, respectively.

### Generation of W_Score and Evaluation of Its Clinical Relevance

**FIGURE 5:**
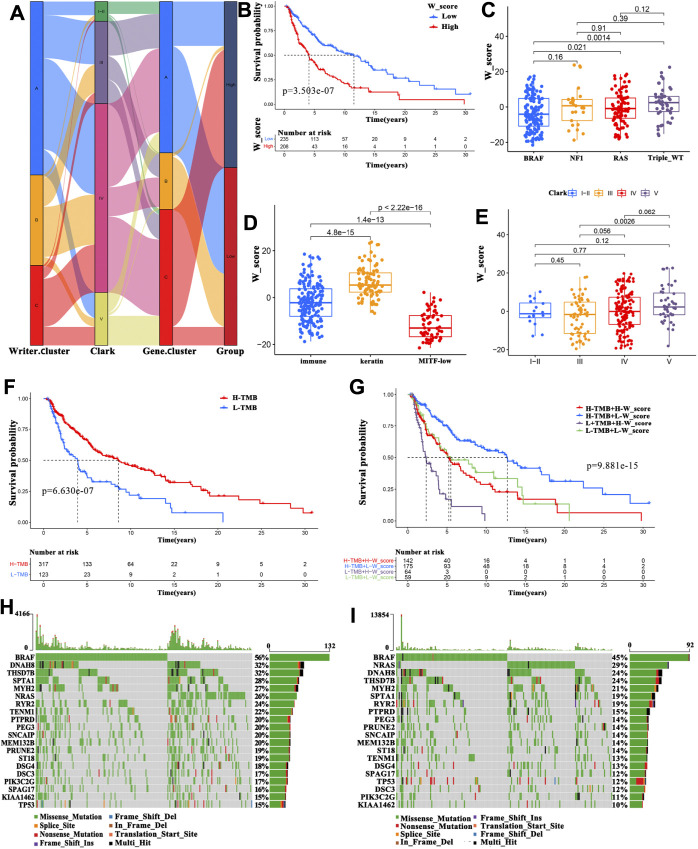
Clinical features associated with the W_Score. **(A)** Alluvial diagram exhibiting the relationship among the writer cluster, Clark level, gene cluster and W_Score. **(B)** Survival analyses for high- and low- W_Score groups in TCGA-SKCM cohort using Kaplan-Meier curves (Log-rank test; *p* < 0.001). **(C–E)** Distribution of W_Score in the different subtypes including gene mutations subtypes **(C)**, immune subclasses **(D)**, and the Clark levels **(E)**. **(F)** Survival analyses for high- and low- TMB groups in TCGA-SKCM cohort (Log-rank test; *p* < 0.001). **(G)** Survival analyses for high TMB and high W_Score (H-TMB + H-W_Score), high TMB and low W_Score (H-TMB + L-W_Score), low TMB and high W_Score (L-TMB + H-W_Score), and low TMB and low W_Score (L-TMB + L-W_Score) patient groups in TCGA-SKCM cohort using Kaplan-Meier curves (Log-rank test; *p* < 0.001). **(H–I)** The waterfall plot of tumor somatic mutation constructed in low- **(H)** and high-W_Score **(I)** groups.

Considering the complexity and heterogeneity of RNA modification, we built a scoring model based on the RNA modification significant genes (950) to quantify the m6A modification pattern of individual patients with SKCM. We discovered that patients with writer cluster C exhibited a high W_Score, whereas those in writer cluster B di not. A similar association also observed between the W_Score and gene clusters (Figures 4E,F and Supplementary Table S5). Figure 5A showed the association among the writer cluster, Clark level, gene cluster, and W_Score. To evaluate the effect of the W_Score on TME, we compared the immune cell infiltration and scores of immune-related signaling pathway between the W_Score-low and -high groups. The results showed that patients with a low W_Score had a relatively high abundance of infiltrating immune cells in the TME (e.g., neutrophils, T follicular helper cells, MDSCs, activated DCs, mast cells, natural killer cells, macrophages, immature DCs, immature B cells, eosinophils, activated B cells, activated CD8^+^ T cells, activated CD4^+^ T cells, and regulatory T cells) (Supplementary Figure S9E) and a significant enhancement in immune activation pathways (e.g., CD8^+^ T effectors pathway, antigen processing machinery pathway, immune-checkpoints pathway, antigen processing and presentation pathway, and JNK and p38 signaling pathway) (Figures 4D,G). Therefore, tumors with low W_Score could be characterized by the immune “hot” phenotype, while high W_Score tumors could be closely linked to immune “cold” phenotype.

To further evaluate the clinical relevance of the W_Score, patients with SKCM were divided into high- and low-W_Score groups based on the cutoff value (0.0291) determined by the “survminer” package. Patients with a low W_Score had a survival advantage (Figure 5B; log-rank test, *p* < 0.001), and the 5-years survival rate was twice than patients in the high-W_Score group (46.1 vs 23.7%, respectively). Interestingly, multivariate Cox regression analysis (gender, age and T-stage, N-stage, M-stage, and clinical stage as covariates) confirmed that W_Score was an independent prognostic biomarker (HR = 1.022 (95% CI = 1.013–1.032), *p* < 0.001; Supplementary Figure S9A). The reliability of the W_Score was verified using 189 patients with SKCM from GEO (GSE65904). Consistent with the above findings, the W_Score-low group displayed a prominent survival benefit (Supplementary Figure S9F; log-rank test, *p* < 0.001), and W_Score could act as an independent prognostic indicator (HR = 1.082 (95% CI = 1.045–1.120), *p* < 0.001; Supplementary Figure S9B). These analyses indicate that W_Score can reflect the RNA modification patterns and be used to predict the clinical outcomes of patients with SKCM.

Subtypes distribution across different tumor grades and stages showed that patients diagnosed as advanced T-stage and higher Clark levels, as well as older ages had an elevated W_Score (Supplementary Figure S9C-D, and Figure 5E), implying the involvement of parameters comprising the W_Score in cancer progression. Based on the most markedly mutated genes, four subtypes were classified in the previous studies, including mutant BRAF, mutant RAS, mutant NF1, and mutant triple-wild-type (triple-WT, lacking hot-spot mutations in BRAF, RAS, or NF1). The BRAF subtype group presented the lowest W_Score (Figure 5C), suggestive of survival advantage, which was in accordance with the previous studies (Network, 2015). Moreover, SKCM patients in the keratin subclass were reported to exhibit worse prognosis than those in the immune subclass and the MITF-low subclass (Network, 2015). As expected, keratin subclass was significantly associated with a higher W_Score and worse prognosis (Figure 5D). These findings demonstrate the reliability of W_Score and augment the previous classification.

Increasing evidence has demonstrated that patients with high TMB have a favorable SKCM prognosis (Edwards et al., 2020; Yan et al., 2020). We also found that patients with a higher TMB displayed a survival advantage (Figure 5F). Therefore, we sought to identify the value of W_Score in evaluating the clinical outcome of patients with TMB. Patients with TMB were divided into four types, including high TMB and high W_Score (H-TMB + H-W_Score), high TMB and low W_Score (H-TMB + L-W_Score), low TMB and high W_Score (L-TMB + H-W_Score), and low TMB and low W_Score (L-TMB + L-W_Score). The patients in the H-TMB + L-W_Score group exhibited the best prognosis, whereas those in the L-TMB + H-W_Score group demonstrated the worst prognosis (Figure 5G). Furthermore, the TMB quantification analysis confirmed that a lower W_Score was remarkably related with a higher frequency of oncogene mutations, such as BRAF (56 vs. 45%), DNAH8 (32 vs. 24%), and DNAH10 (24 vs. 17%) (Figures 5H,I). These data enabled us more comprehensively to understand the effect of W_Score classification on genomic variation and prognosis of patients with SKCM, as well as to reveal the potential roles of RNA modification in the individual somatic mutations.

### Potential Role of W_Score in Predicting Chemotherapeutic Drugs and Response to Immunotherapy With a PD-L1 Blocker

**FIGURE 6:**
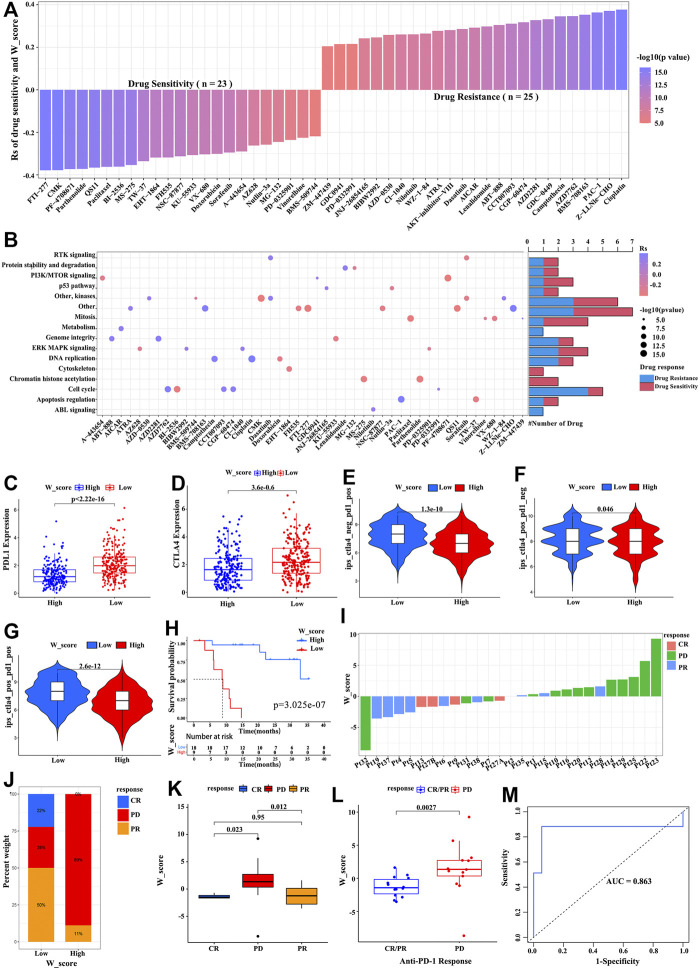
The W_Score predicting the drug sensitivity and immunotherapy efficacy. **(A)** The association between W_Score and drug sensitivity (Spearman analysis). **(B)** Signaling pathways targeted by drugs that are resistant (red) or sensitivity (blue) to the W_Score. **(C,D)** Differences in expressions of PD-L1 and CTAL-4 between high- and low- W_Score groups (Wilcoxon test; *p* < 0.0001). **(E–G)** The correlation between co-expression of PD-L1 and CTLA-4 and the W_Score (samples with SKCM from TCGA), including CTAL-4 positive and PD-1 negative (ips_ctla4_pos_pd1_neg) **(E)**, CTAL-4 positive and PD-1 positive (ips_ctla4_pos_pd1_pos) **(F)**, and CTAL-4 negative and PD-1 positive (ips_ctla4_neg_pd1_pos) **(G)**. **(H)** Kaplan-Meier curve showing overall survival of SKCM patients between high- and low- W_Score groups in the anti-PD1 immunotherapy cohort (GSE78220 cohort; Log-rank test; *p* < 0.001). **(I)** The relationship of W_Score with clinical response to PD-1 blockade immunotherapy (GSE78220 cohort). **(J)** The proportion of patients with response to anti-PD-1 immunotherapy in high and low W_Score groups (GSE78220 cohort). PD, progressive disease; CR, complete response; PR, partial response. **(K,L)** The differences in the W_Score among distinct PD-1 blockade immunotherapy response groups (GSE78220 cohort). **(M)** The predictive value of W_Score in SKCM patients treated with anti-PD-1 immunotherapy (GSE78220; AUC, 0.863).

To further explore the effects of the W_Score on drug response, we evaluated the correlation between the W_Score and the response to drugs. A total of 48 drugs were identified from the GDSC database (Figure 6A). Among them, the sensitivity of 25 drugs was associated with the W_Score, including the farnesyltransferase inhibitor FTI-277 (Rs = −0.38, *p* = 6.72E-17), the WNT/β-catenin pathway inhibitor FH535 (Rs = −3.20, *p* = 9.07E-12), and the AKT and ERK inhibitors Sorafenib (Rs = −0.30, *p* = 2.93E-10), whereas 23 drugs showed the resistance related to the W_Score, including the DNA double-strand break and apoptosis activator Cisplatin (Rs = 0.38, *p* = 1.08E-16), the AKT inhibitor VIII (Rs = 0.28, *p* = 2.11E-09), and the cyclin-dependent kinase inhibitor CGP-60474 (Rs = 0.38, *p* = 1.01E-11). Then, the signaling pathway enrichment analysis based on drug-targeted genes revealed that the W_Score was linked to drug-associated signaling pathways, including the PI3K/MTOR signaling pathway, RTK signaling pathway, ERK/MAPK signaling pathway, p53 signaling pathway, and cell cycle signaling pathway (Figure 6B).

Immunotherapies based on CTLA-4/PD-1/PD-L1 inhibitors have achieved astounding clinical efficacy in malignant tumor therapy. Significant efforts have been made to identify biomarkers for predicting immunotherapy response, such as the expression of PD-L1 and CTLA-4 (Van Allen et al., 2015; Gibney et al., 2016; Miao et al., 2018). As shown in Figures 6C,D, the expression of PD-L1 and CTLA-4 obviously elevated in the low W_Score group. Subsequent analysis also revealed that a low W_Score was remarkably associated with high expression of PD-L1 and/or CTLA-4 (Figures 6E–G). Given that the W_Score was strongly related to the immune microenvironment, we further evaluated the power of the W_Score model in predicting patients’ responses to immunotherapy with PD-1/PD-L1 inhibitors, based on the anti-PD-1 cohort (GSE78220; metastatic melanoma) and anti-PD-L1 cohort (IMvigor210; advanced urothelial cancer). Patients with a lower W_Score displayed remarkably clinical benefits and a significantly prolonged survival in both cohorts (Figure 6H; *p* < 0.001, and Supplementary Figure S9G; *p* < 0.001). The better therapeutic outcomes and improved clinical response to anti-PD-1/L1 immunotherapy in W_Score-low patients were confirmed (Figures 6I–L, and Supplementary Figure S9H-I). Moreover, an AUC value with 0.870 indicated that the quantification of RNA modification patterns was a robust biomarker for evaluating patients’ prognosis and response to immunotherapy (Figure 6M). Taken together, these analyses imply that the W_score model could provide the selection of chemotherapeutic drugs and contribute to evaluating the response to anti-PD-1/L1 immunotherapy in SKCM.

## Discussion

Mounting evidence has demonstrated that RNA modifications have an essential role in innate immunity, inflammation, and antitumor activity by interacting with multiple “writers” (Lin et al., 2020; Shulman and Stern-Ginossar, 2020; Chen et al., 2021; Chong et al., 2021). While current research has mainly focused on one type of RNA modification “writers”, such as m1A “writers” and m6A “writers” (Zhang et al., 2020; Gao et al., 2021), the roles of numberous types of RNA modification “writers” in tumor progression have not been fully recognized. Therefore, in this study, we explored global changes in four types of A-associated RNA modification “writers” (m6A, m1A, A-to-I, and APA) at the molecular level and the relationship between their interaction and the TME, so as to identify distinct RNA modification patterns in the tumor immune microenvironment. This would eventually reinforce our understanding of antitumor immune response and aid in the development of efficient immunotherapy strategies for patients with SKCM.

In this work, three distinct RNA modification patterns were identified based on 26 RNA modification “writers”. These three modification patterns showed remarkably different tumor immune microenvironment characteristics. The writer cluster B was characterized by the immune activation and abundant lymphocyte-infiltration, which correspond to the immune-inflamed phenotype. The writer cluster C was characterized by the immune-desert phenotype. The writer cluster A was characterized by the infiltration of immune cells together with the activation of TGF-β signaling pathway, JAK/STAT signaling pathway, and WNT signaling pathway, which correspond to the immune-excluded phenotype. The activated TGF-β pathways may suppress immune function by preventing lymphocytes from entering the tumor parenchyma, while specific molecular inhibitors that target TGF-β can restore the antitumor immunity by remodeling the tumor immune microenvironment (Fabregat et al., 2014; David et al., 2016; Mariathasan et al., 2018; Panagi et al., 2020). Studies have shown that the immune contexture of the TME influences the development and progression of SKCM, and can be used to predict immunotherapeutic response and prognosis (Galon and Bruni, 2019). The levels of tumor-infiltrating immune cells (e.g., natural killer cells, DCs, macrophages, and CD4 + /CD8 + T cells) are associated with the immune response that is generated (Topalian et al., 2016; Galon and Bruni, 2019; Zeng et al., 2020). Thus, these findings demonstrate the reliability of immunophenotypic classification based on distinct RNA modification patterns.

Furthermore, GO analysis and univariate Cox regression analysis suggested that prognosis-related DEGs could act as the signature genes of A-associated RNA modification. Of note, similar to the classification results of the RNA modification “writers”, the three gene categories based on A-associated RNA modification signature genes were constructed and were found to be remarkably related with different clinical outcomes and immunophenotypes. This confirmed that the significance of RNA modification “writers” in shaping the different landscape of TME. Because of the heterogeneity in RNA modification, we built a W_Score model to quantify the RNA modification patterns of individual patients. The RNA modification pattern characterized as the immune-inflamed phenotype displayed a lower W_Score, whereas those the modification patterns characterized as immune-desert and immune-excluded phenotypes presented a higher W_Score. We also found that the W_Score was significantly associated with clinical features of patients with SKCM and tumor subtypes. Noticeably, the W_Score was shown to be an independent prognostic biomarker for patients with SKCM, and this finding was verified in the GEO-SKCM cohort.

Because the benefits of survival and therapeutic responses of patients subjected to tumor immunotherapy remain limited to a small population, classifying patients to obtain better insight into the optimal use of tumor immunotherapy is an effective strategy (Harel et al., 2019; Albittar et al., 2020; Guo and Shen, 2020). Patients with SKCM were divided into high- and low-W_Score groups based on the cutoff value, and these two groups displayed extremely distinct tumor immune microenvironment characteristics. The low W_Score exhibited an immune “cold” phenotype characterized by a lack of immune-cell infiltration, indicative of a non-inflammatory TME (Kim and Chen, 2016). The high W_Score exhibited TME characteristics that were opposite to those of the immune “hot” phenotype, which was characterized by an enhanced immune-cell infiltration, indicative of an inflammatory tumor immune microenvironment (Turley et al., 2015; Chen and Mellman, 2017). These two groups could represent the distinct potential mechanisms modulating tumor immune escape, corresponding to different treatment strategies. The “hot” tumor (low W_Score group) exhibited a favorable immune-activated phenotype and may be responsive to ICIs (Wang et al., 2021). Notably, our data revealed that low W_Score showed a significantly high TMB. Analysis of genomic data indicated that a high TMB induced an increase in the number of neoantigens and boosted the immune response rate to ICI therapies (Liu et al., 2019; Hodi et al., 2021). In addition, patients with low W_Score had high enrichment scores in the immune-related signaling pathways. Roper et al. discovered that high expression of genes with antigen-processing machinery was beneficial for checkpoint immunotherapy (Roper et al., 2021). Ahn et al. reported that the PD-1 blockade promoted the activation of CD8^+^ T effectors and resulted in faster clearance of infection (Ahn et al., 2018). Furthermore, the clinical benefit of checkpoint immunotherapy is associated with high expression of immune-checkpoint proteins and suppression of angiogenesis (Castet et al., 2019; Zavareh et al., 2021). Of note, we confirmed that patients with low W_Score in two immunotherapy cohorts exhibited an enhanced immune response and long-term clinical benefits of immunotherapy. These analyses indicated that the W_Score could be regarded a powerful “predictor” for identifying patients sensitive to immunotherapy. In particular, the W_Score also has predictive values to evaluate the benefits of chemotherapy or targeted therapies. RNA modification patterns determined by the interaction of RNA modification “writers” were linked to immune phenotypes and affected the therapeutic effects of ICIs, which may provide clues on appropriate drug and immunotherapy strategies for SKCM.

In this study, we comprehensively analyzed RNA modification patterns based on four types of A-associated RNA modification “writers”, and revealed the association between these RNA modification patterns and tumor immune microenvironment characteristics. We also constructed a W_Score model and identified their clinical utility in targeted therapy and tumor immunotherapy. This paper has some limitations that further experimental verification are needed to validate these findings. This study thus highlighted the clinical significance of the crosstalk of multiple A-associated RNA modification. We believe that the findings of this study will contribute to developing personalized immunotherapy strategies for patients with SKCM.

## Data Availability

The datasets presented in this study can be found in online repositories. The names of the repository/repositories and accession number(s) can be found in the article/Supplementary Material.

## Supplementary Material

Supplementary Figure S1 Overview of study design.

Supplementary Figure S2 Mutation characteristics of 26 A-related RNA modification “writers” in TCGA-SKCM cohort. **(A)** The mutation co-occurrence and exclusion analyses for 26 RNA regulators. Co-occurrence, blue; Exclusion, Khaki. **(B)** The prognostic analyses for 26 RNA regulators base on univariate Cox regression model. **(C)** GSVA enrichment analysis showed the differences between “writers” mutation and non-mutation tumors.

Supplementary Figure S3 CNVs regulating the “writers” expression in TCGA-SKCM cohort. *P* value less than 0.05 indicated a statistical difference.

Supplementary Figure S4 The characteristics of tumor microenvironment in three RNA modification patterns. Evaluating the abundance of each tumor-infiltrating immune cell in three writer clusters using ssGSEA (GSE65904). (**p* < 0.05; ***p* < 0.01; ****p* < 0.001).

Supplementary Figure S5 Association between tumor immune microenvironment and 26 A-related RNA modification “writers”. **(A)** The correlation between the abundance of tumor-infiltrating immune cells and RNA regulators (TCGA-SKCM cohort; Spearman analysis; **p* < 0.05; ***p* < 0.01; ****p* < 0.001). **(B)** Differences of immuneScore between WTAP high expression and low expression groups (Wilcoxon test; *p* < 0.001). **(C)** Differences of tumor-infiltrating immune cells between WTAP high expression and low expression groups. (**p* < 0.05; ***p* < 0.01; ****p* < 0.001). **(D)** Differences in the expression of adhesion molecules, MHC molecules, and costimulatory molecules between WTAP high expression and low expression groups. (**p* < 0.05; ***p* < 0.01; ****p* < 0.001). **(E)** Differences in immune-activated pathways between WTAP high expression and low expression groups. (**p* < 0.05; ***p* < 0.01; ****p* < 0.001). **(F,G)** Kaplan-Meier curve showing overall survival of SKCM patients between high- and low- WTAP expression groups in the anti-PD-1 immunotherapy cohort (GSE78220 cohort) **(F)**, and GEO-SKCM cohort (GSE65904) **(G)**.

Supplementary Figure S6 Unsupervised clustering of 26 A-related RNA modification “writers” in the TCGA-SKCM cohort. **(A–D)** Consensus matrices of the TCGA-SKCM cohort for k = 2–5. **(E)** The difference in expression of 26 RNA regulators among the writer cluster A, writer cluster B, and writer cluster C. (TCGA-SKCM cohort; **p* < 0.05; ***p* < 0.01; ****p* < 0.001). **(F)** 2281 differently expressed genes DEGs genes shown in venn diagram.

Supplementary Figure S7 Immune-related molecular characteristics in distinct gene clusters. **(A–D)** Unsupervised clustering of prognosis-related differently expressed genes (DEGs) in the TCGA-SKCM cohort (k = 2–5). **(E)** Evaluating the abundance of each tumor-infiltrating immune cell in three gene clusters using ssGSEA. (**p* < 0.05; ***p* < 0.01; ****p* < 0.001). **(F)** Difference in immune-related pathways among three gene clusters. (**p* < 0.05; ***p* < 0.01; ****p* < 0.001). **(G–I)** Difference in the immune-activation **(G)**, chemokines and cytokines **(H)**, and immune-checkpoint **(I)** related gene expression among three gene clusters. (**p* < 0.05; ***p* < 0.01; ****p* < 0.001).

Supplementary Figure S8 The expression of 26 “writers” in three gene clusters (GSE65904). **(A)** The survival curves of three gene clusters (GSE65904; Log-rank test; *P* < 0.05). **(B)** The differences in expression of 26 A-related RNA modification “writers” in three gene clusters. (GSE65904; ANOVA analysis; **p* < 0.05; ***p* < 0.01; ****p* < 0.001).

Supplementary Figure S9 The correlation between W_Score and clinicopathological parameters. **(A,B)** Multivariate Cox regression analysis for W_Score in TCGA-SKCM cohort **(A)** and GEO-SKCM (GSE65904) cohort **(B)** shown by the forest plot. **(C,D)** Difference in W_Score among distinct clinical subgroups [age **(C)** and T-stage **(D)**] in TCGA-SKCM cohort. (**p* < 0.05; ***p* <0.01; ****p* < 0.001). **(E)** Evaluating the abundance of each tumor-infiltrating immune cell between high- and low- W_Score groups using ssGSEA. **(F)** Kaplan-Meier curve showing overall survival of SKCM patients between low- and high- W_Score groups (GSE65904) (Log-rank test; *P* < 0.001). **(G)** Kaplan-Meier curve showing overall survival of patients between low- and high- W_Score groups in the anti-PD-L1 immunotherapy cohort (IMvigor210 cohort; Log-rank test; *P* < 0.001). **(H)** The differences in the W_Score among distinct PD-L1 blockade immunotherapy response groups (IMvigor210 cohort). **(I)** The proportion of patients with response to PD-L1 blockade immunotherapy in high and low W_Score groups (IMvigor210 cohort). PD, progressive disease; CR, complete response; PR, partial response; SD, stable disease.

## Abbreviations

3′UTR, 3′-untranslated region; APA, Alternative polyadenylation; A-related RNA modifications, adenosine-related RNA modifications; CNV, Copy number variations; DCs, Dendritic cells; DEGs, Differentially expressed genes; GDSC, Genomics of Drug Sensitivity in Cancer; GEO, Gene Expression Omnibus; GSVA, Gene Set Variation Analysis; GTEx, Genotype-Tissue Expression; HRs, hazard ratios; ICIs, immune-checkpoint inhibitors; ROC, receiver operating characteristic; SKCM, skin cutaneous melanoma; ssGSEA, Single-sample gene set enrichment analysis; TCGA, The Cancer Genome Atlas; TCIA, The Cancer Immunome Database; TME, tumor microenvironment.
