# Supplementary figures and images for: Crosstalk Between Four Types of RNA Modification Writers Characterizes the Tumor Immune Microenvironment Infiltration Patterns in Skin Cutaneous Melanoma
Source: Front Cell Dev Biol. 2022 Jan 26;10:821678. doi: 10.3389/fcell.2022.821678 (PMC8826580; doi:10.3389/fcell.2022.821678)

**Figure S1**

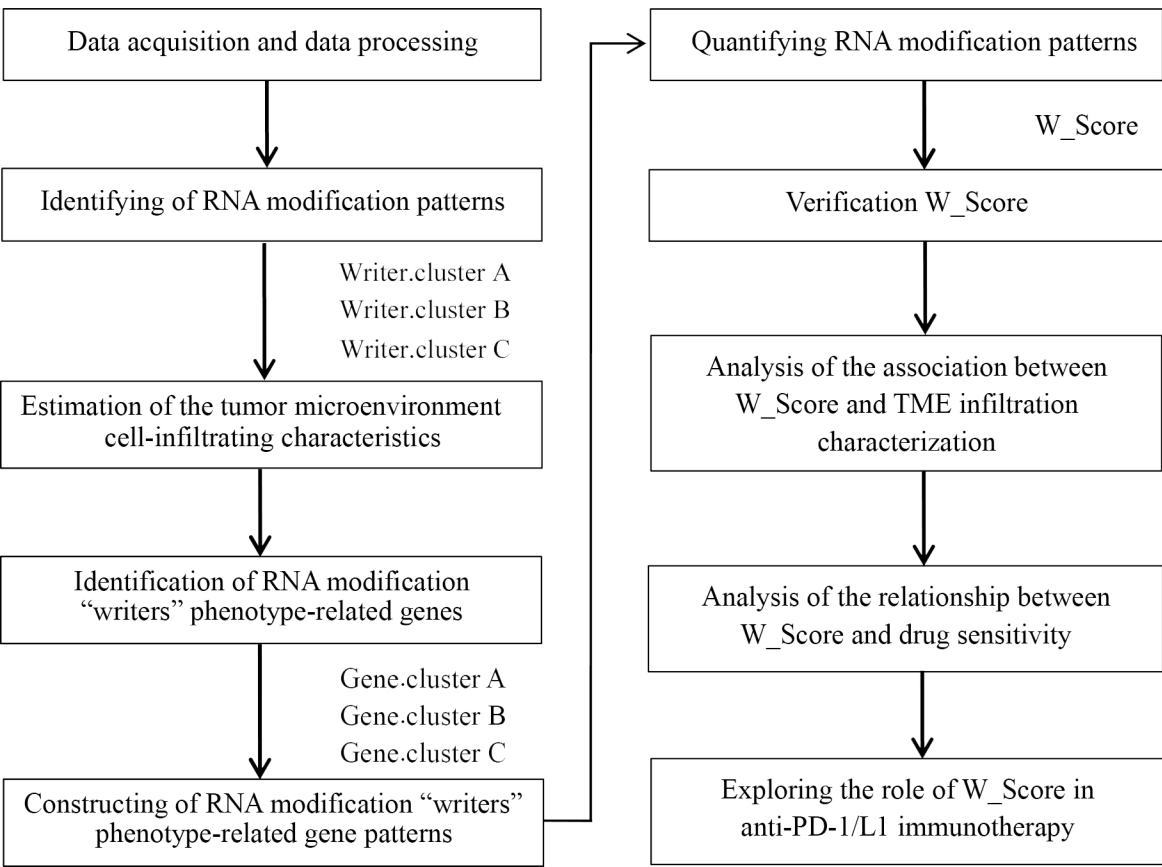

Figure S2

A

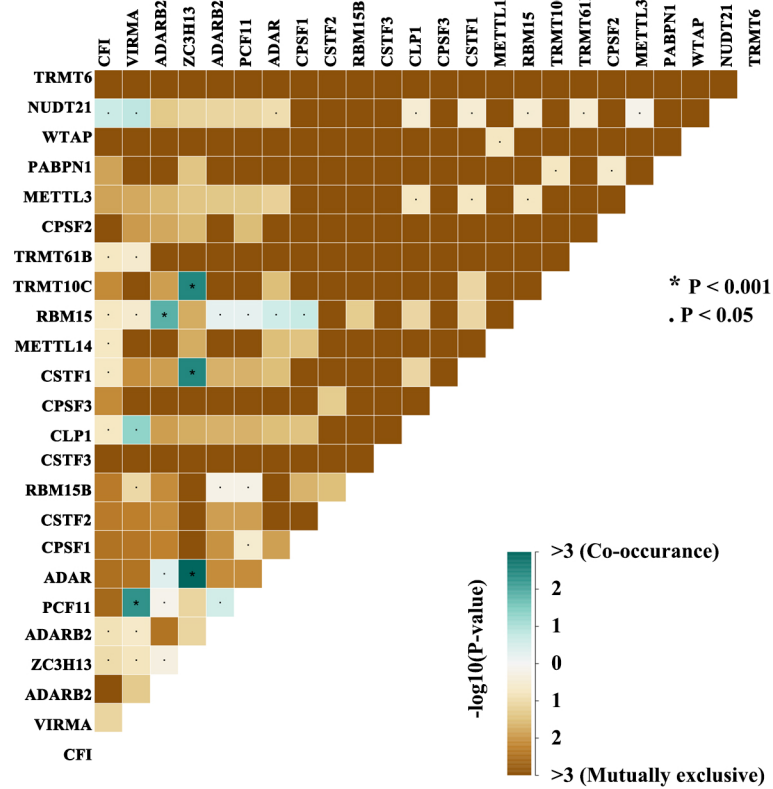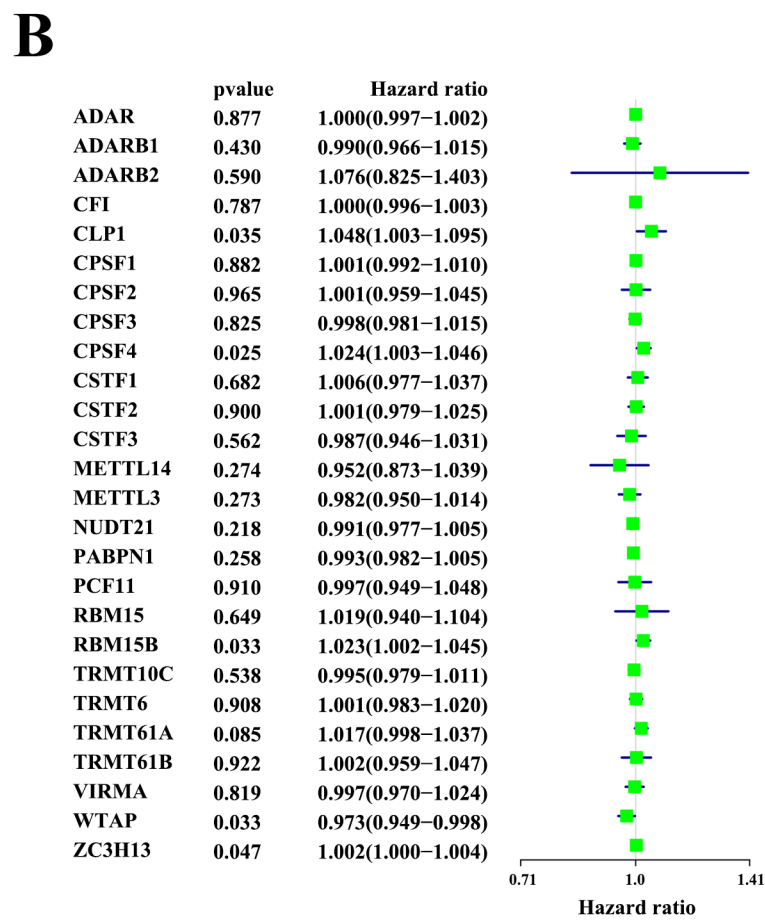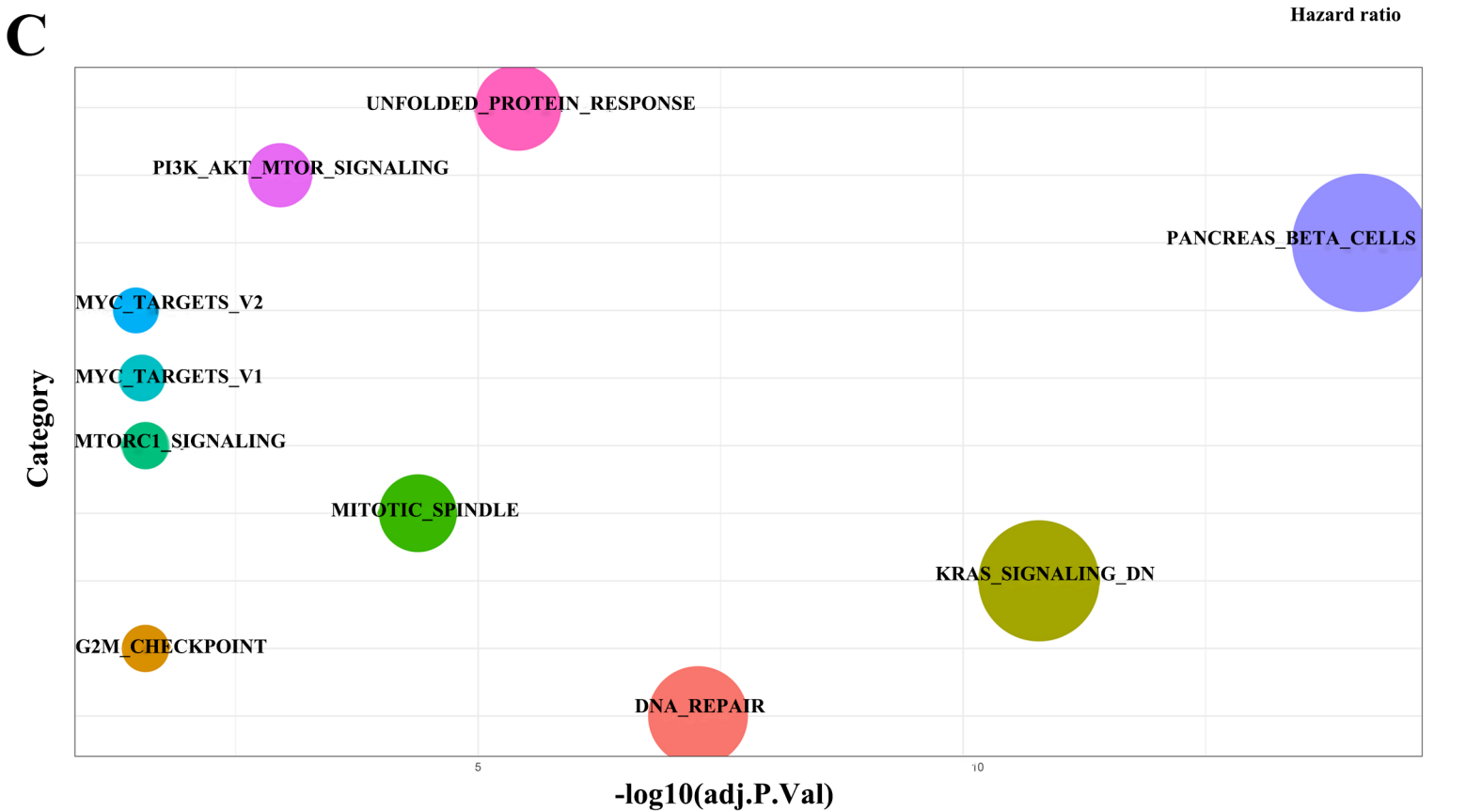

Figure S3

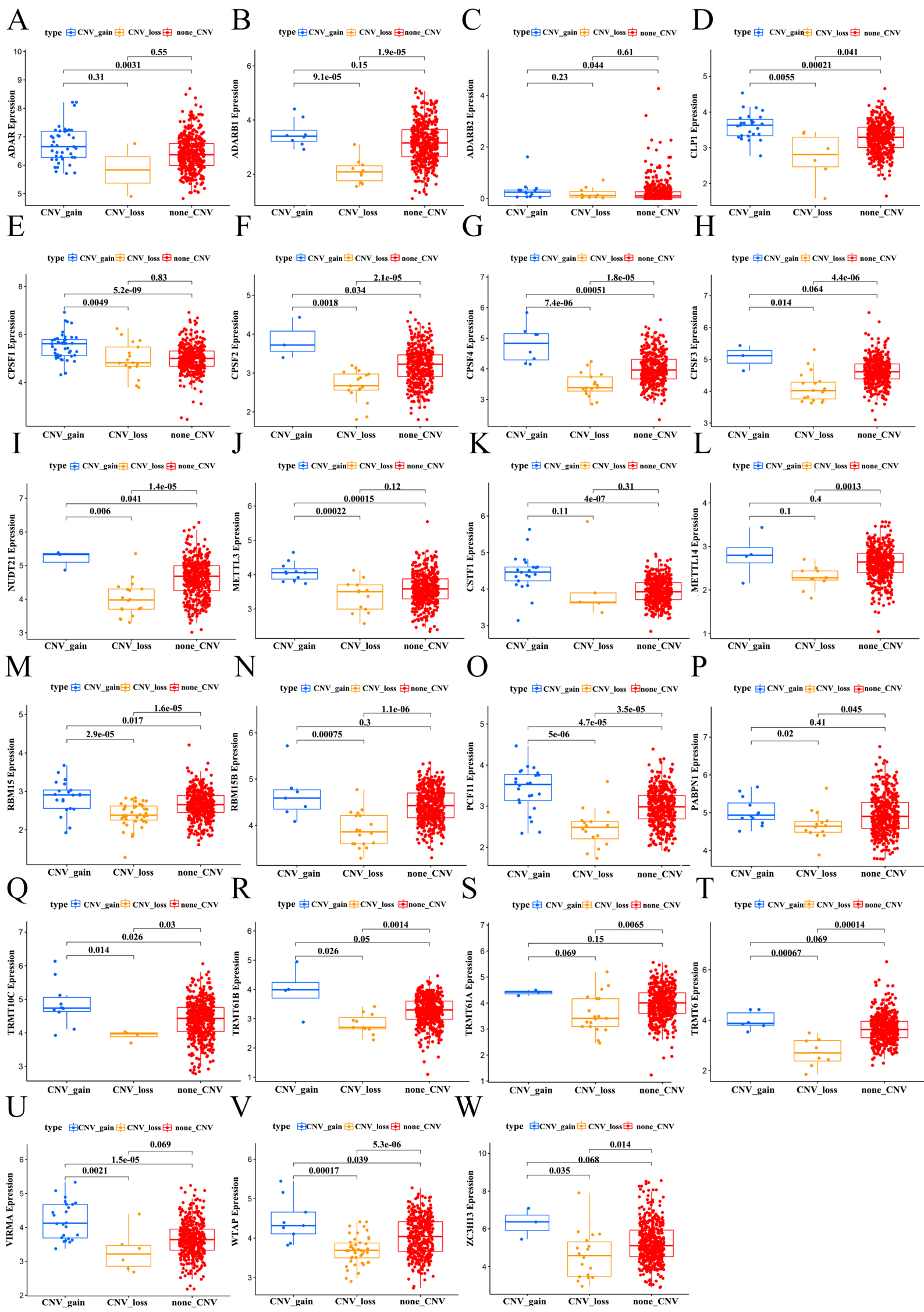



A

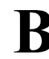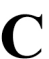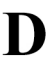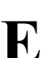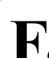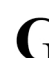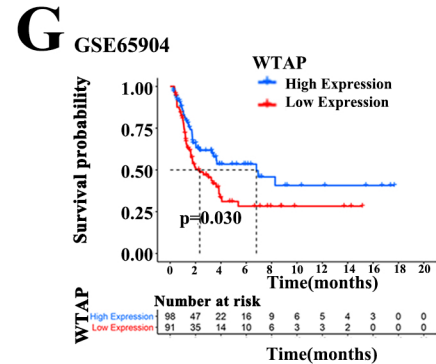

Figure S6

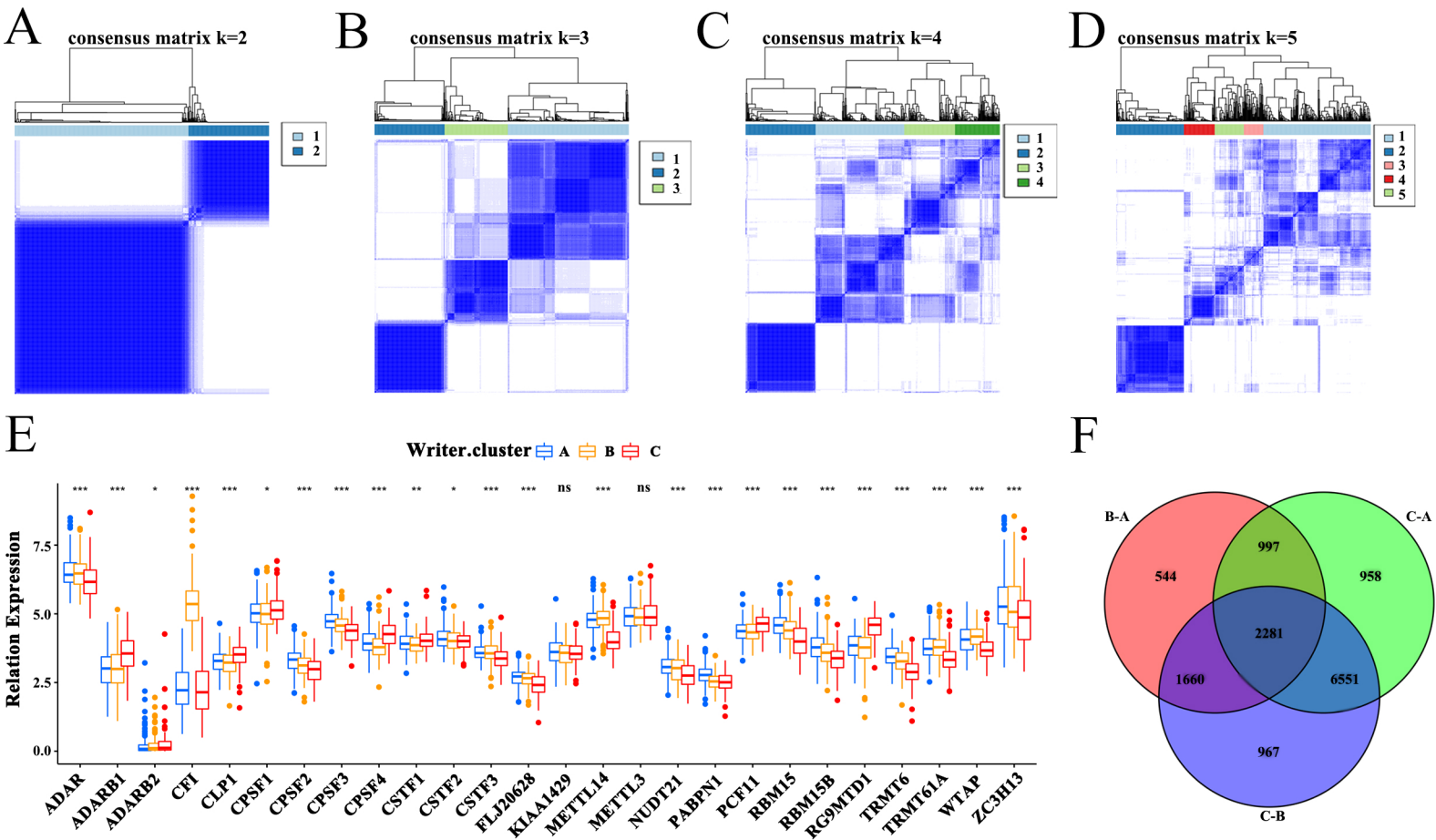

Figure S7

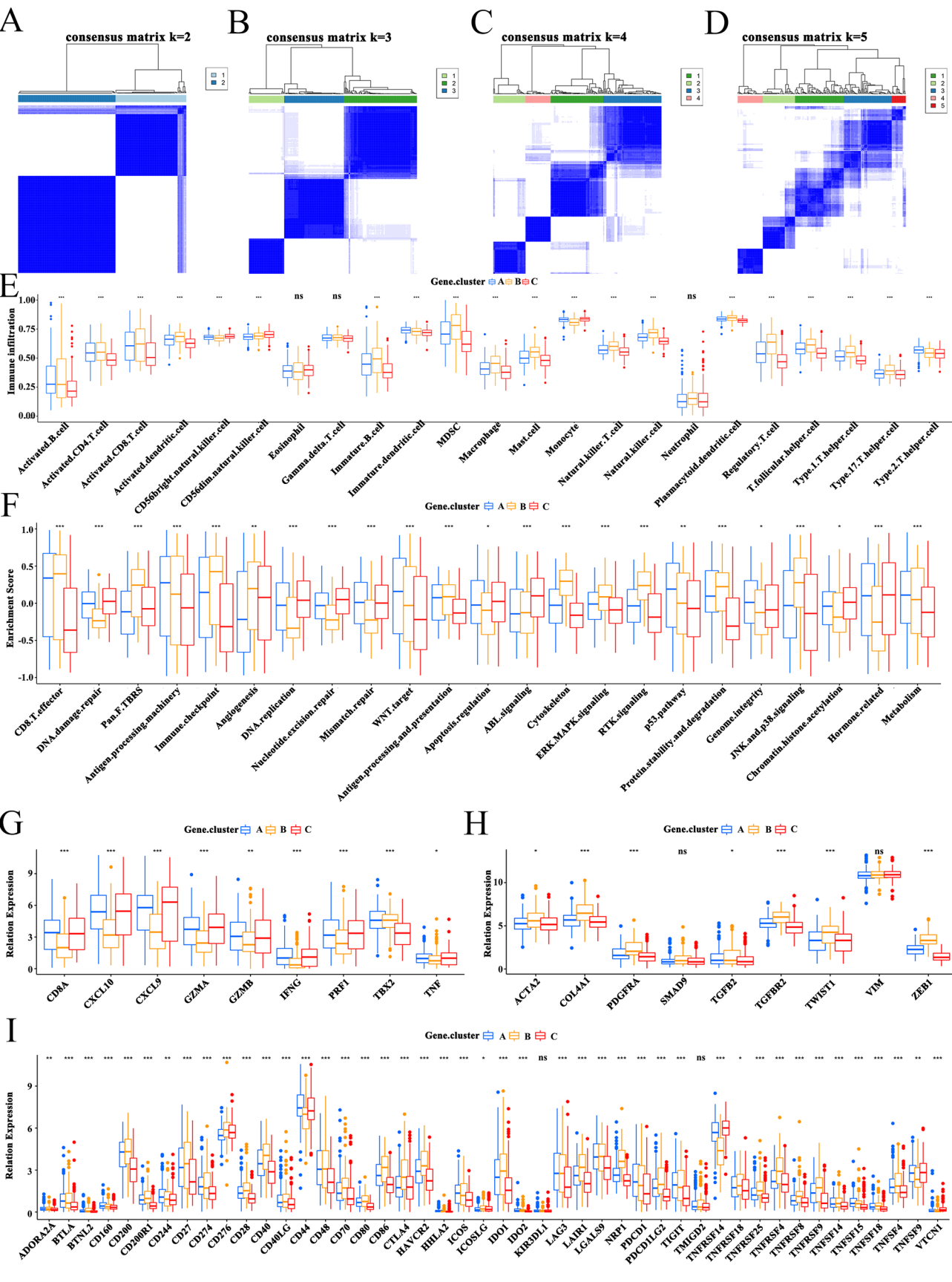

Figure S8

A

GSE65904

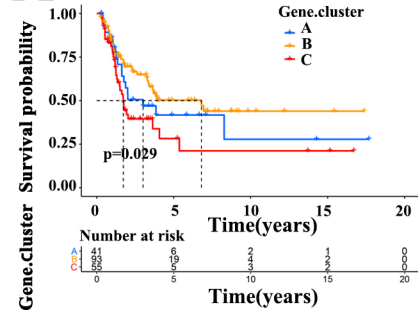

B

GSE65904

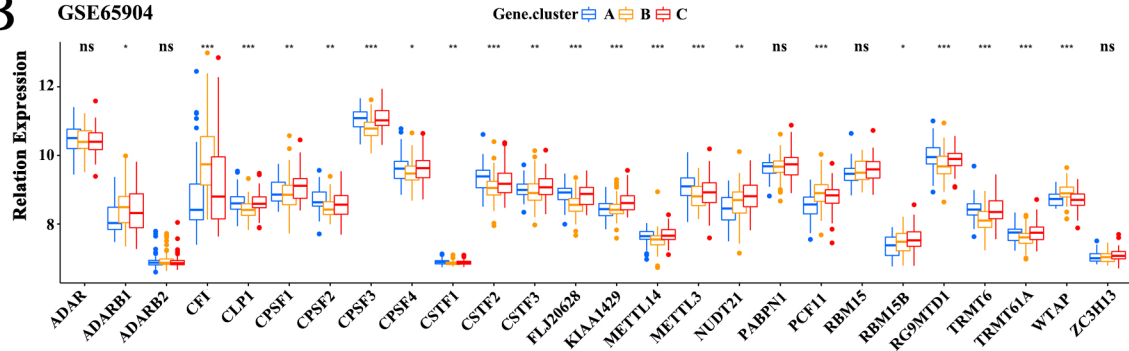

Figure S9

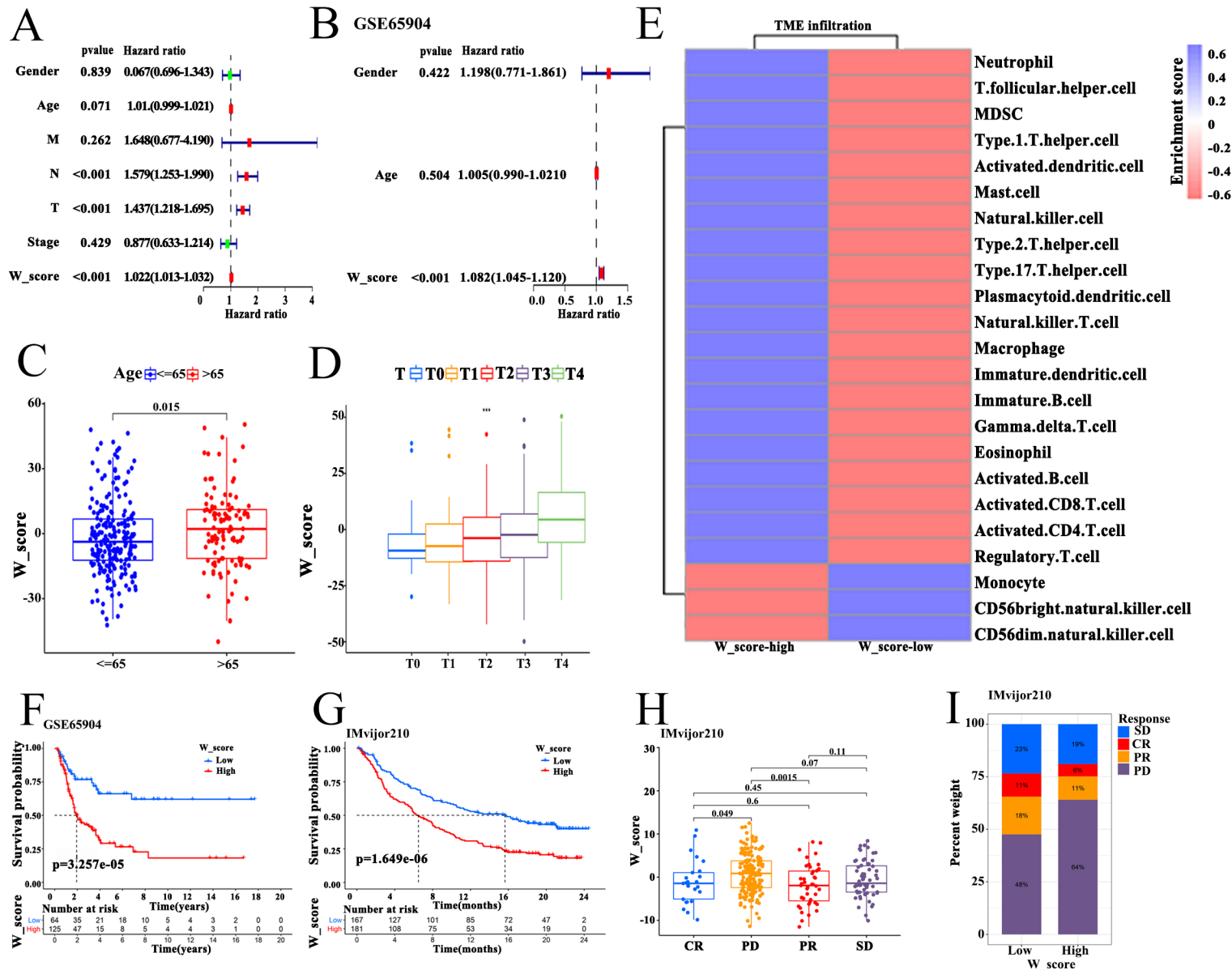

Supplement: Supplementary file 1 [file DataSheet1.PDF]
